# Supplementary material for: Are carbon emissions trading and green financial instruments synergistic? -Comprehensive quantitative research based on content analysis
Source: PLoS One. 2024 Mar 7;19(3):e0298601. doi: 10.1371/journal.pone.0298601 (PMC10919601; doi:10.1371/journal.pone.0298601)
Supplement: S1 Data — (ZIP) [file pone.0298601.s001.zip › Data/地级市Z_F工作报告.zip!/║┌┴·╜¡/╣■╢√▒⌡╩╨/└┤╫╘╣1⁄2╓┌║┼í╛┬φ┐╦╩2╛▌═°í┐_2019.docx]

各位代表：

现在，我代表市人民政府向大会报告工作，请予审议，并请市政协各位委员提出意见。

一、2018年工作简要回顾

过去一年，是全市实施“十三五”规划承上启下的重要一年。在省委、省政府和市委的坚强领导下，在全市上下的共同努力和广大市民的大力支持下，我们坚持以习近平新时代中国特色社会主义思想和党的十九大精神为指导，全面深入持续贯彻落实习近平总书记对我省重要讲话精神，学习贯彻落实习近平总书记在深入推进东北振兴座谈会上的重要讲话和考察黑龙江的重要指示精神，牢固树立“四个意识”，坚定“四个自信”，坚决做到“两个维护”，积极应对经济下行压力和挑战，把握稳中求进工作总基调，践行新发展理念，统筹推进“五位一体”总体布局，协调推进“四个全面”战略布局，按照战略考虑、系统安排、重点突破、全面提升的工作原则，扎实做好稳增长、促改革、调结构、惠民生、防风险各项工作，保持了全市经济社会平稳发展。预计全年地区生产总值比上年增长5.5%左右；一般公共预算收入完成384.4亿元，增长4.4%；税收占财政收入比重提高到84.4%。

——现代农业迈出新步伐。种植业品质持续提升，绿色有机食品认证面积超过1000万亩，“三品一标”认证数量达1791个，分别占全省的14.3%和23.2%。高标准农田、水稻节水灌溉面积分别提高到610万亩和300万亩，增长13.5%和10%；尽管粮食生产受到春季干旱、夏季洪涝、秋季台风等各类自然灾害影响，总产仍实现300亿斤。新型农业经营体系不断完善，新增农民合作社规范社107个，组织域外农产品展销会13场，新办“村村网店”1667家，覆盖率超过90%。农产品加工转化率达到65%。农村改革持续深化，率先在全省完成村级清产核资、国有林场“三定”改革任务，232个行政村建立集体资产股份合作制，农业农村内生活力进一步增强。

——工业经济注入新动能。发挥供给侧结构性改革和创新驱动引领支撑作用，大力改造升级“老字号”、深度开发“原字号”、培育壮大“新字号”，工业生产稳中向好、速度效益双提升，全市规模以上工业增加值和利税分别增长5.5%和6.5%左右。开复工市重点战略性新兴产业项目54个，完成投资占工业项目的42%，高于上年8.9个百分点，信息技术、高端装备制造等战略性新兴产业增加值占规上工业比重提高到24%左右。科技成果转化“市场导入”试点启动实施，新增市级科技企业孵化器18家、高新技术企业163家，高技术产业增加值增长16%。落实产业配套对接、各类要素引领扶持、兑现承诺政策等措施，帮助企业解困突围、降本增效、提档升级。全年为企业降低税费、用地、用工等成本89.6亿元，市企信担保中心为中小微企业贷款担保额达150亿元，开发建设的龙哈工业云注册企业超过500家，55户临规企业成长为规上企业。市属国企改革“1+N”政策体系初步形成，列入混改计划的115户市国资委出资企业已完成25户，清理处置“僵尸企业”29户。哈空调通过“三项制度”改革扭亏为盈、闯出新路。

——现代服务业释放新活力。国家服务业综合改革试点城市建设扎实推进，预计第三产业增加值对经济增长的贡献率超过70%。贯彻落实“绿水青山就是金山银山，冰天雪地也是金山银山”理念成效明显，旅游供给和节会水平不断提升，冬夏两季旅游持续升温，全年共接待国内外游客8567万人次，过夜游客平均在哈停留3天、人均消费超过3300元，旅游总收入达1376亿元、增长16.9%。太平机场进出港旅客首次超过2000万人次，总量和增量均居东北首位。全力打好防范化解重大风险攻坚战，强化金融市场监管，坚决遏制风险增量，及时处置风险存量，系统规范地方政府性债务源头管控，金融乱象整治取得实效。坚持防范和发展并重，积极适应国家政策要求，建立鼓励金融业发展政策，金融业主要营业收入超过850亿元，增长5%。跨境物流通道更加便捷，哈尔滨至洛杉矶货运包机航线实现首航，哈尔滨至莫斯科公铁联运实现常态运营，综保区跨境电商平台自去年8月运营以来实现1.8万单。组织开展国际广告节、哈洽会、哈科会等活动203个，会展业直接收入达23.6亿元、增长10%。

——哈尔滨新区建设和对外合作取得新成效。坚决落实省委、省政府要求，顺利完成市委机关搬迁，呼兰利民开发区“六街一镇”交由新区托管，推动重大公共设施和公共服务向新区倾斜、事权向新区集中，省级第二批确定下放事权254项，市级一次性下放全部事权284项，滨北公铁两用桥、阳明滩大桥北部连接线正式通车，江北一体发展区、江南政策协同区互动发展，全力推动“一江居中、南北互动、两岸繁荣”格局的形成。借鉴自贸区和其它国家级新区改革试点经验，全面推行负面清单管理模式，“最多跑一次”“证照分离”试点等104项改革接力突破，一批改革成果在省市得到推广。区域合作进一步深化，102个深哈合作项目投产运营14个，在建33个，完成投资133.8亿元，深哈共建产业合作园区落实并启动；哈（哈尔滨）长（长春）协同发展合作框架协议正式签署，两市合作互动开启新征程。招商引资规模持续扩大，签订亿元以上项目302个，实际使用内资1658亿元、外资36.5亿美元。对外贸易呈现新变化，民营企业进出口额增长18.7%、总量首次超过国有企业，对俄进出口额增长51.3%，首届中国国际进口博览会我市展内成交额占全省98%。

——省会城市建设取得新成果。启动新一轮城市总体规划编制，完成供水、排水、供热、热电联产、垃圾处理等专项规划，中心城区实现“多规合一”。重大基础设施建设有序推进，哈佳快速铁路、哈牡客专如期开通，哈尔滨火车站改建一新，太平国际机场T2航站楼建成投用，地铁1号线三期调试运行，城乡路高架桥竣工通车，新建改造城乡道路470公里、供热供水排水燃气网源厂站19座、老旧管网253公里。转变传统交通组织理念，系统整治城市交通拥堵，采取渠化改造平交路口、维修平整区街巷路、设立单行道、缩减地铁施工占地、严查交通违法行为、清退违规出租车、上线智慧交通系统、建立完善规章制度等措施，交通拥堵状况初步改善。重拳整治城市乱象，拆除违法非农设施221个，整改超标看护房981个，“大棚房”问题专项整治全面告捷，露天烧烤、趸船码头、非法采砂等得到有效治理。周密部署打好污染防治攻坚战，综合施策治理垃圾围城、内河水系、黑臭水体、空气污染等顽疾。及时关闭向阳垃圾处理场，启动阿城玉泉和松北呼兰垃圾焚烧厂规划建设，双城垃圾焚烧厂一期和6个县乡垃圾处理站建成投用。完成城市内河、松花江污染源普查和整治规划，实施相关工程45项，清理沿河污染源1146处，实现阿什河消除劣五类水质年度目标，磨盘山、松花江等市级水源地清理整治任务全面完成，松花江干流哈尔滨段水质总体优良。淘汰改造燃煤锅炉1191台，更换生物质锅炉1万余台，更新双燃料出租车1270台、新能源公交车1323台，秸秆综合利用和露天禁烧工作强力推进，环境空气质量优良天数达310天，比上年增加40天，创有PM2.5监测以来最好水平，位列全国169个重点城市空气质量改善程度第一名。取缔市区高尔夫球场，拆除围栏改建成开放式湘江公园、还绿于民，新建荷和园、东直园等公园游园20个，城区新植树木9.8万株、新增绿地242公顷，人工造林10.5万亩，成功入选全球首批“国际湿地城市”。

——人民生活获得新实惠。预计城乡居民人均可支配收入均增长6.5%。惠民行动承诺如期兑现，完成投资超过130亿元。举全市之力开展精准脱贫攻坚战，巴彦、木兰2个省级贫困县脱贫摘帽，60个贫困村脱贫出列，贫困发生率由0.98%下降到0.46%。尽最大努力保障人民生活，城镇新增就业10.22万人，登记失业率3.76%；城乡居民基本养老金待遇、最低生活保障标准进一步提高，基本医疗保险实现全面融合；新增养老床位3871张；新建残疾人康复、托养及助残服务站130家。对城区59个、涉及2.6万户的棚改历史遗留项目进行逐一梳理、分类施策、全面落实解决，已具备安置回迁条件居民约1万户，到2020年全部完成安置；基本建成棚改住房8394套，新开工建设7154套；全力解决历史遗留的359个项目26.5万户居民不动产权证办理问题，已有25.8万户具备办证条件；改造农村危房6358户、室内厕所1万户；城区1万余户居民“吃水难”问题得到解决，农村剩余3472个村屯饮水安全工程全面完成。创建国家级校园足球、省级冰雪特色学校76所，城区中小学校“百万青少年上冰雪”实现全覆盖，建设中小学校及托幼机构“阳光食堂”798个；成功举办东亚文化之都哈尔滨年等活动，国家文化消费试点合作单位由城区覆盖到所有区县（市）；取消药品加成政策由公立医院扩大到所有专业公共卫生机构，贫困人口9种大病救治率由60%提高到100％；哈尔滨籍运动员平昌冬奥会获得奖牌数占中国代表团的三分之一。加强社会治理和应急管理，妥善处置“8·25”重大火灾事故，整改火灾隐患14.6万处，打掉涉黑涉恶犯罪集团和团伙59个，全市食品监督抽检合格率达99.7%，中央巡视组交办信访事项合理合法部分化解率达98.8%，信访总量下降21%，社会总体保持和谐稳定。

——政府自身建设得到新加强。始终把学习贯彻落实习近平新时代中国特色社会主义思想作为首要政治任务，把不折不扣贯彻落实党中央各项决策部署作为重大政治责任，中央政治巡视、中央环保督察“回头看”反馈意见整改取得初步成效，政府公职人员践行“四个意识”“两个维护”的自觉性进一步增强。始终把深化政府改革作为整顿作风优化环境的根本举措，工业和仓储项目审批最短只需25个工作日，新建商品房不动产权登记办结时限由20个工作日缩减到2个工作日，“最多跑一次”事项达90%以上，走在全国前列；企业开办时间减少到最多5个工作日，低于国务院规定标准3.5个工作日，企业集群登记新模式被国务院通报表扬；向城区下放市级行政职权和公共服务事项167项，全面取消13类社区证明的做法被民政部在全国推广；坚持新官理旧账，依法整治政府失信事项392件；建立民营企业家座谈会商、外出培训等服务新机制，推动民营企业主动对接市场、强化内部管理、增强发展活力。全市新登记市场主体14.5万户，增长33.8%。为企业和群众提供优惠公共法律服务12项。自觉接受人大、政协监督，人大代表建议、议案和政协委员提案办结率均达100%。加强对政府公共资金、公共资源、国有资产的梳理、规范、监管和审计，促进增收节支18.2亿元。

双拥和预备役、民族宗教、妇女儿童、统计监测、机关事务、普法、气象、人防、地震、档案、供销社、地方志、红十字等工作也都取得了新进展。

各位代表，成绩来之不易，这是习近平新时代中国特色社会主义思想科学指引的结果，是党中央、国务院、省委、省政府和市委坚强领导的结果，是历届市政府接力奋斗的结果，是市人大、市政协和社会各界监督支持的结果，是全市人民团结一致、努力拼搏的结果。在此，我代表市人民政府，向全市人民，向人大代表、政协委员、各民主党派、各人民团体、社会各界人士以及离退休老同志，向中省直单位和驻哈解放军指战员、武警官兵，向心系家乡的哈尔滨人，向所有关心支持哈尔滨振兴发展的同志们、朋友们，致以崇高的敬意和衷心的感谢!

在肯定成绩的同时，我们也清醒地看到，发展不平衡不充分问题仍然十分突出。主要表现为经济总量小、发展速度慢，部分指标没有完成年初预期。农业产业链短、价值链处于低端；工业产业结构单一，传统产业拉动力减弱，新兴产业发育迟缓，转方式、调结构任务繁重而紧迫；服务业层次较低、有效供给不足；体制机制短板明显，部分领域改革仍然没有破题，市场在资源配置中的决定性作用发挥还不到位，营商环境有待优化，国企内生动力不足，民营经济发展缓慢，解放思想、改革创新的任务仍很繁重；开放进展不快、步伐不大，对经济增长的贡献率持续过低；城市现代治理体系不健全，污染防治、交通治堵和供水、排水、供热、物业管理、教育、医疗、文化等民生工作，与满足和保障百姓生活、城市发展需求还有很大差距；一些政府公职人员的作风能力素质还不适应改革发展要求，工作系统性统筹性协调性不够，发展措施不聚焦不聚力，有效应对新产业新业态新需求能力尚显不足，拼劲不够，动力不强，专业化水平不高，不担当不作为甚至乱作为现象依然存在，“人民城市人民爱”的城市主人翁精神还有待大力弘扬。对此，我们要在今后的工作中，切实采取有效措施，认真加以解决。

二、2019年主要任务

2019年是新中国成立70周年，是全面建成小康社会关键之年，是推动高质量发展、实现全面振兴全方位振兴的重要一年。新的一年，机遇与挑战并存，机遇大于挑战。从不利因素看，外部环境趋紧、内生动力不足、市场竞争加剧等带来的经济下行压力仍将持续，补齐体制机制、经济结构、开放合作、思想观念四个方面短板，尚需一个艰难过程，特别是思想不解放、观念滞后、市场意识不强、传统思维定式和路径依赖，仍是制约我市振兴发展的根本和要害。加之生态保护、基础设施、民生事业等各类历史和现实矛盾问题叠加，政府工作面临着巨大压力。但我们更要看到，当今世界百年未有之大变局，丰富了我国重要战略机遇期新内涵，为全市抢抓宏观政策逆周期调节机遇、推动补短板强弱项快发展提供了难得的有利条件。特别是党中央、国务院高度重视支持东北振兴，并作出系列决策部署。党的十八大以来，习近平总书记两次主持召开东北振兴座谈会并作出重要讲话，三年两次亲赴黑龙江考察指导工作，把脉定向、开出良方、给出良策，为我们推动新时代振兴发展注入了坚定信心和强大动力。省委、省政府高度聚焦支持省会城市建设，为我市更好发挥对全省的辐射功能、外溢效应和带动作用，提供了有力的政策支持。哈尔滨资源富集，产业基础坚实，经过长期努力，创新驱动潜能、工业转型升级潜能、文化旅游时尚生态产业潜能等正加速释放，重大基础设施支撑作用正逐渐显现，尤其是随着哈佳快速、哈牡客专等高铁线路通车运营，以哈尔滨为中心的一至两小时经济圈基本形成，集聚辐射人口近4000万，为全市发展带来了广阔的消费市场空间。面对新机遇新挑战新要求，只要我们辩证看待国际环境和国内条件的变化，认真贯彻落实“巩固、增强、提升、畅通”八字方针，秉持战略考虑、系统安排、重点突破、全面提升工作原则，进一步突出问题导向，坚持有所为有所不为，依据形势、市场、需求变化和资源禀赋、区位条件、产业基础等优势，实事求是，与时俱进，把解放思想落实在具体行动上，形成推动振兴的有效机制和办法，瞄准方向、保持定力，扬长避短、扬长克短、扬长补短，一以贯之、久久为功，以人人尽责、人人共享，不做局外人、不当旁观者的城市精神，撸起袖子加油干，哈尔滨的明天一定会更加美好！

按照市委十四届五次全会部署，今年政府工作指导思想是：以习近平新时代中国特色社会主义思想和党的十九大精神为指引，持续深入贯彻落实习近平总书记对我省重要讲话、在深入推进东北振兴座谈会上的重要讲话和考察黑龙江的重要指示精神，贯彻中央经济工作会议精神，按照省委、省政府和市委决策部署，以解放思想为先导，坚持稳中求进工作总基调，坚持新发展理念，坚持推动高质量发展，坚持以供给侧结构性改革为主线，坚持深化市场化改革、扩大高水平开放，加快建设现代化经济体系，继续打好“三大攻坚战”，统筹推进稳增长、促改革、调结构、惠民生、防风险工作，进一步转变作风，打造营商好环境，促进投资增长，激发内生动力，壮大发展新动能，加快推动哈尔滨全面振兴全方位振兴，以优异成绩庆祝新中国成立70周年。

主要预期目标是：地区生产总值增长5.5%以上；一般公共预算收入增长3.5%；城乡居民人均可支配收入与地区生产总值同步增长。着重做好以下工作：

（一）以优化营商环境为基础，全面深化改革。全面深化改革是新时代哈尔滨振兴发展的治本之策、关键一招。我们要拿出自我革命的勇气和胸怀，坚定改革信心，做实改革举措，释放改革活力，提高改革效能。

优化营商环境。结合新一轮政府机构改革和事业单位改革，建立健全并严格执行优化营商环境考核评价体系。持续推进“强市简政、强区扩权、强街固网”改革，推广项目承诺制试点，推进“不见面审批”“最多跑一次”，探索“容缺审批”和重点发展区域环评能评安评统筹新机制；优化各级职能权责配置，动态管理下放事权，解决多头管理、上下交叉、管理空白等问题，实现每件事都有部门负责，一件事只在一级办理；推动城市管理、市场监管等领域执法力量下沉到街镇，逐项明确具体情形的行政裁量标准和办结时限，打造高效便捷的政务环境。加强法治政府建设，完善社会信用体系，鼓励采取更多措施建立承诺制，加大对失信人的联合惩戒力度，依法兑现政府承诺，打造诚实守信的人文环境。强化市场在资源配置中起决定作用落实机制，推动政府部门工作重心转向加强事中事后监管，更多运用分类监管、“双随机一公开”等方式，打造公平公正的市场环境。强化要素保障，启动电力体制综合改革试点，优先安排存量土地落项目，提高土地集约利用水平和供地效率。整合出台新一轮人才政策，强化教育、医疗、住房、康养等服务保障，给予平台、资金、政策、环境等方面支持，让域内人才安心安身安业，让引进人才在哈尔滨扎根立业、尽展才华。

激发国有企业改革发展活力。坚持把混合所有制改革作为深化国企改革的突破口，加快引进外部投资者，鼓励管理层和骨干员工持股、具备条件的企业进入资本市场融资。完善市属国企现代企业制度，抓好董事会建设和市场化选聘职业经理人，激发内在潜力。加快处置“僵尸企业”。深化国有资产管理体制改革，组建国有资本运营公司、投资公司，调整优化国有资本布局，规范资本运作，提高国有资本回报。将市属国企闲置不动产和商业区、大学等周边机关事业办公用房，配置到工业、服务业和创业孵化等能够产生效益的领域。

毫不动摇鼓励支持引导非公有制经济发展。扎实推进东北地区民营经济发展改革示范城市建设，建立与民营企业对话沟通常态机制，坚持定期通报、征求意见、专题会议、走访调研、联络直通、定向培训六项制度，零障碍、高效率为民营企业提供精准服务。落实各级各类市场准入和减税降费等降成本政策措施，保障中小微企业平等获得市场要素、参与市场竞争。引导金融机构扩大信贷规模，确保新增贷款向民营企业、中小微企业倾斜，做强政策性担保机构，努力解决民营企业融资难、融资贵问题。大力弘扬企业家精神，切实增强亲商爱商富商意识并创造良好氛围，构建“亲”“清”新型政商关系。

（二）培育壮大新动能，推动经济高质量发展。发展是硬道理。我们要坚定不移向经济建设这个中心聚焦发力，不断深化对市情的认识，以市场为导向，以构建现代产业体系为目标，以提升一产价值链、打造二产新增长点、增强三产供给能力和水平为切入点，推动产业融合、产业升级、产业发展，形成多点支撑、多业并举、多元发展的产业格局。

坚持项目牵动和投资拉动并重，存量生成与增量引进同步，切实增强经济发展后劲。把产业项目建设作为实现振兴发展的突破口，启动产业项目提质增效三年攻坚战，强化统筹协调和服务保障，完善考核激励机制，集中力量抓好一批竞争力强、带动能力大、对地方贡献突出的好项目大项目，全力扭转产业项目投资下滑趋势。全年实施市重点产业项目200个以上。科学规划全市产业主攻方向，绘制多规合一产业功能区地图，围绕市场需求和资源禀赋、产业定位、产业链等谋项目引项目落项目，形成主导产业和优势产品。抓住国家基础设施领域补短板的窗口期，围绕智慧城市、民生公益保障、铁路公路机场等基础设施建设，持续扩大有效投资，拉动经济增长。推动政府投融资体制改革，激发民间投资活力，更多引进外部资金，更好发挥政府投资的引导作用和放大效应。

坚持以农业供给侧结构性改革为主线，加快构建现代农业产业体系、生产体系、经营体系，通过提升农产品市场价值，倒逼农业结构调整和降低生产成本，实现农业增效、农民增收。落实藏粮于地、藏粮于技，新建高标准农田70万亩以上，粮食产能稳定在300亿斤以上，为保障国家粮食安全、端牢中国饭碗做出贡献。扩大杂粮杂豆蔬菜等经济作物种植；提高生猪养殖加工产业链延伸水平，提升乳制品质量效益和市场竞争力，发展特色水产养殖业；因地制宜发展森林养殖、森林种植、森林旅游、森林食品，做强做大食用菌、中药材、大榛子等林中经济。加快五常大米等域内独特地理标识品牌体系建设。强化市场营销，对接国内一二线城市中高端消费市场，推进直供直销基地建设，构建种植加工销售均衡受益的利益分配机制；推广私人定制、互联网众筹等营销新模式，开展“交邮合作”，支持农村客运带货配送，解决农村电商“最后一公里”问题。突出市场导向，加快机械化水利化科技化进程，加大引导土地规模经营和推进农业“三减”力度。推动绿色农产品种植、精深加工和乡村旅游等三次产业融合发展，让农业成为有看头、有赚头、有奔头的产业。

坚持以存量带增量、创新促升级，推动工业强市战略深入实施，着力支持大企业产业带动、中小微企业集聚发展。发挥智慧城市建设对工业转型升级和产业融合的牵动作用，按照资源统筹、高效利用、开放共享、适度超前的原则，引进战略投资者，年内建成首批十大云平台，并面向社会推出一批智能生产、智慧管理、智享生活等领域的应用需求，促进产业技术与生产快速对接、产品及时升级、管理成本有效控制，提升企业市场竞争力。建设国家级大数据创新创业基地。建立完善中小企业与军工企业、大学、科研院所对接机制，加快培育引进既懂设计又懂工艺、既懂技术又懂市场的复合型人才，在军民融合发展上实现新突破。建立企业诊断中心，扩大科技成果市场导入规模，支持引导科研单位和企业组建中俄等中外联合技术创新中心，着重解决企业管理组织能力不强、创新动力不足、技术储备不够等问题。加大财政支持，鼓励校所科技人员和大学毕业生围绕市场需求领办创办科技企业，推动形成科技企业发展氛围和产业基础。大力发展科技金融，强化对科创企业多种形式融资服务，促进科技企业从小到大、由弱变强。全年新增科技型中小企业1000户，高新技术企业150家以上。

坚持补齐供给短板与引入外部需求协同发力，加快释放服务业发展潜能。夏季旅游重点突出生态之城、音乐之城、时尚之城、历史文化名城题材，提升高雅艺术、群众文化、欧陆风情、山林穿越、电子竞技、汽车营地、南病北治、生态观光养生等旅游供给能力和水平，打造全国一流的避暑胜地。冰雪旅游重点扩大冰雪运动、冰雪体验、冰雪文化娱乐、冰雪服饰和装备供给，启动规划建设世界顶级冰雪主题乐园和大亚布力景区，面向长三角、珠三角、东南亚等地区开展针对性市场营销，打造全国及国际地区性冰雪旅游首选目的地。强化政策导向，系统推进中央大街、中华巴洛克、花园街等历史街区整体性改造建设及业态布局，持续推出一批吃住行游购娱特色旅游产品和新业态，探索建设旅游康养体系，进一步发展城市夜经济，增强消费对经济增长的拉动力。加强旅游基础设施建设，依托文化旅游云平台建设，强化旅游市场监管和服务，完善“先行赔付”等机制，真正让游客来得放心、玩得舒心。建设人社云平台，着力提升产业发展人力要素保障功能，引进一批国内外知名人力资源服务企业。落实国家产融合作试点政策，推动金融业收入向千亿元目标迈进。

（三）科学统筹精准施策，构建开放合作高地和协调发展新格局。开放合作、协调发展是实现全面振兴全方位振兴的必由之路。哈尔滨是因开放而发展起来的城市，我们必须提高站位，放宽视野，互通有无，相互借力，不断开拓协同发展新局面。

加大对外开放力度。深度融入“一带一路”和“中蒙俄经济走廊”建设，着力打造对俄日韩欧美开放门户，把哈尔滨与生俱来的开放传统转化为经济优势。加强对俄经贸、农业等多领域合作，协力推进哈欧、哈俄班列和哈绥俄亚陆海联运通道常态化运营，扩大俄罗斯商品进口。加强与日韩及东南亚在食品加工、旅游等领域交流合作，加大对欧美等国家和地区招商引资、技术合作力度，推动开通哈尔滨至美国安克雷奇货运航线。加速综保区与内陆港一体化进程，探索一区多园模式，力争取得食用水生动物、肉类、整车进口等口岸功能，不断提高通关便利化。配合做好机场二期扩建工作，保障第二跑道按期开工建设。以跨境电商综合试验区建设为重点，系统推进临空经济区基础设施、产业项目建设和配套服务。扩大与国际友城、世界文都城市友好往来，推动对外交流合作向科技教育文化体育人才等更多领域、更高层次拓展。

推动区域合作向纵深发展。发挥深哈合作园区示范引领作用，全面导入深圳产业生态体系、政策制度和管理经验，加快推进两地资源互补、产业互联、人才互动、信息互通。推动哈（哈尔滨）长（长春）城市群规划衔接，落实协同发展合作框架协议，促进两市务实合作实现新突破。加强省会城市建设，强化要素供给、功能配套和产业互动，为省内城市协同发展提供科学引领和高效服务。按照全市产业功能区布局，完善重点园区、城区东部及东南部等水电气热基础设施配套条件，推动城区聚焦中高端产业、县域聚焦特色资源开发，加快形成区县经济优势互补、分工协作、错位发展新格局。

统筹推进城乡一体化发展。围绕乡村振兴战略，统筹规划，分步实施，扎实走好城乡融合发展之路。落实产业兴村强县行动，坚持一乡一业、一业多乡、一村一品、一品多村，用特色小产品抢占市场大份额，提升乡村自我“造血”功能和县域经济实力。全面完成农村集体产权整市推进改革任务，探索农村集体经营性建设用地入市改革，让农民共享改革红利。集中开展农村人居环境整治专项行动，统一规划布局，形成村屯收集、乡镇转运、区域集中处理的村镇垃圾收运处理体系，改造室内洁净厕所4万个，推动农村环境实现更大改善。

（四）标本兼治长效防控，坚决打好“三大攻坚战”。这既是全市现实需要，更是我们肩负的重大责任使命。我们必须抓重点、补短板、强弱项，不折不扣落实好党中央重大决策部署，为全市发展守住底线。

坚决打好防范化解重大风险攻坚战。全面加强和规范政府性债务管理，确保新增债务符合政策及城市发展要求、旧债务不发生系统性风险。紧盯金融领域重大风险，加强小额贷款、融资担保等方面的风险摸底排查和舆情监测预警，完善应对预案，严厉打击经济领域特别是金融领域违法犯罪，做好防范和处置非法集资工作，强化对人民群众树立防范金融风险意识的宣传教育，控制风险增量。

坚决打好精准脱贫攻坚战。坚持扶贫与扶志扶智相结合、专业行业社会扶贫相补充，抓好责任、政策和工作落实，加大对深度贫困村财政、金融、社会保障等支持力度，完成延寿县脱贫摘帽、17个村脱贫出列年度任务，力争10个区县（市）贫困人口全部退出。着重抓好产业扶贫，做细做实如订单农业、庭院经济等扶贫产业，通过以购代捐、消费扶贫等方式解决贫困户农产品初期销售问题。巩固提高“千医万户健康扶贫”连心工程实效，减少和防止贫困人口返贫，确保脱贫质量。

坚决打好污染防治攻坚战。贯彻“绿水青山就是金山银山，冰天雪地也是金山银山”理念，完成中央环保督察“回头看”年度整改任务。严控燃煤总量和质量，因地制宜解决棚户区、城郊镇（乡）村燃煤散烧问题，通过压块、翻埋还田、生物质热电联产等方式，有效探索秸秆综合利用路径，继续抓好秸秆露天禁烧工作，落实工业减排限排要求，加强汽车尾气、餐厨油烟、工地扬尘等治理，有效改善环境空气质量。落实河湖长制，重点推进阿什河、何家沟、马家沟、信义沟、银水湾等城区水系综合治理，进一步根治城市黑臭水体，对沿河污染源数据信息定位落图、挂图作战、开展专项整治，加强排水系统收集，优化污水处理厂布局，加快推进22个重点镇污水处理设施建设，确保松花江干支流国控断面优良比率达到考核要求、沿岸环境根本改善。着力解决城区垃圾围城问题，启动实施玉泉固废综合处理园区和松北呼兰、双城、双琦等垃圾焚烧厂新建和扩建工程，推进向阳、月亮湾、哈东地区等垃圾场存量垃圾治理，开展生活垃圾分类示范创建和袋装直运试点，整治城区废品回收站无序乱象，建设废旧家电回收分解中心，启动污泥处理设施建设，餐厨垃圾集中收运无害化处理实现主城区全覆盖、医疗垃圾实现城乡全覆盖，城区生活垃圾无害化日处理能力提高到5000吨以上。围绕争创国家园林城市，持续推进沿街补绿、拆墙透绿、景区扩绿，城区新植树木3.5万株，植树造林7万亩。强化湿地、山体和黑土地保护，做好生态环境修复。建设环保云平台，全面提升污染防治和环境保护智能化管理水平，让天蓝山绿水清的良好生态成为哈尔滨最普惠的民生、最具竞争力的发展优势。

（五）着力推动哈尔滨新区建设，加快实现一江居中、南北互动、两岸繁荣的发展格局。新区建设既是国家战略，也是牵动哈尔滨乃至黑龙江振兴发展的关键引擎。我们必须紧扣“三区一极”战略定位，按照省委、省政府和市委的部署要求，集中资源、协同发力，推动新区加快建设，充分释放发展活力。

建设体制机制新高地。以更先进的理念、更开放的姿态、更坚定的决心，汇百家之长，走创新之路。立足打造“龙江特区”，支持新区复制自贸区经验，有效承接省市下放事权，加快推进相对集中行政许可权改革和行政审批标准化建设，深化区街管理一体化改革，努力实现居民办事不出街、企业办事不出区，着力打造营商环境最优的国家级新区。推动新区在人力资源管理、研发机构落地、创新创业、招商引智、科技成果转化等方面创新政策，更好发挥先行先试、示范引领作用。

打造产业发展新引擎。突出高端化产业发展导向，促进高端装备、生物医药等产业集群发展。抓好科技创新、金融商贸、文化旅游三大产业功能区建设，充分盘活成熟地块和闲置楼宇，着力引进一批金融机构、企业总部、众创空间和高新技术企业，争取更多国家和省重点实验室、重点产业项目、中外联合技术创新中心落户新区。坚持新区对俄合作与深哈合作整体推进、联动发展，加快建设哈尔滨松北（深圳龙岗）科技创新产业园。

提升宜居宜业新品质。高标准高水平完成新区总体规划修编，科学确定功能分区和建设时序。启动新区基础设施建设三年行动计划。推动公共基础设施项目和公共服务资源向新区倾斜，谋划启动建设国际冰雪运动中心、会展中心和文化艺术城项目，规划建设新区师大附中学校、新区市少年宫，加快建设市妇儿医疗中心。加强街区精细化管理，引导带动江南江北人口、经济要素均衡配置、协调发展，把新区打造成为宜居之城、创业之城。

（六）更加致力补齐民生领域短板，让人民群众共享振兴发展成果。人民对美好生活的向往，就是我们的奋斗目标。我们要顺应民心，尊重民意，关注民情，严格控制和压减一般性财政支出，从严安排“三公”经费预算，把财政资金更多用在保障民生上。重点抓好30件民生实事。

（1）畅通城市路网，完善二环高架体系，打通三环交通堵点和二环三环西部联络线，推进跨城区内河断头路桥梁建设,启动实施机场第二通道、三棵树跨线桥、三环路王岗地道桥等项目，按照路平灯亮树绿标准全面维修改造区街巷路。（2）加快推进地铁2号线一期、3号线二期工程，启动4号线、5号线一期工程，确保1号线三期载客运营。（3）系统治理交通拥堵，统筹优化平交路口交通组织和道路单行设置，升级改造交通信号灯岗700处，安装LED交通引导屏45块。将城市公交车、出租车、渣土车、环卫作业车等全部纳入智慧交通云平台管理。全面取缔无路权车辆，重拳整治三环以内和三环至四环之间主干街路非法营运三轮车、残疾人代步车。（4）提升公交服务功能，新增新能源公交车500台，调整开辟公交线路20余条。（5）推进供热网源互联互通，启动智慧供热试点，提高城市供热安全性稳定性。（6）有效治理燃煤锅炉，淘汰不具备升级改造条件的35吨及以下锅炉，启动65吨及以上锅炉节能和超低排放改造。（7）开展物业管理年活动，强化管理服务能力提升，着力解决城区800余万平方米弃管小区物业管理问题。启动既有住宅加装电梯和老旧小区改造。（8）拓展市民休闲空间，规划建设整治江北沿江公园和新区友谊公园，新建武源街、向阳路等15个公园和游园，拆除儿童公园围栏，免费开放太阳岛公园，建设3处夏季城区江滨浴场。（9）解决棚改历史遗留问题，安置逾期未回迁居民4000户，基本建成棚改住房6000套，新开工2000套。加大“烂尾楼”依法整治力度。（10）维修改造主城区二次供水设施，解决老城区6.9万户居民“吃水难”问题。加大对磨盘山等水源地保护力度，启动城区应急供水水源及处理厂新建和升级改造。（11）依托城管云平台建设，整顿商业街区乱象，规范早晚夜市，取缔室外占道经营，治理牌匾广告混乱无序、低水平罗列状况。（12）保障农村五保户和低收入家庭住房，改造危房7000户,剩余6000户明年全部完成。（13）建设“四好农村路”200公里，改造危桥20余座。（14）持续稳定就业，安排1.3亿元失业保险基金，开展援企稳岗护航行动，推动大学毕业生、农民工、退役军人等重点群体就业创业，抓好对残疾人、戒毒康复人员等弱势群体、特殊群体的就业创业服务。全市新增城镇就业9万人，登记失业率控制在4.3%以内。（15）发展素质教育，创建足球特色学校50所、冰雪特色学校（幼儿园）60所，建立市和区县（市）少年宫上下联动发展格局。（16）优化学校网点布局，闲置校舍优先支持学前教育、职业教育和优质民办教育发展。（17）加大对公办和普惠性民办幼儿园的财政支持，解决托幼机构供给不足、质量不优、收费过高等问题。（18）促进优质教育资源均衡配置，启动普通高中“市区共建”试点，有序开展区、县（市）域内义务教育校长教师交流。（19）强化师德师风建设，建立完善教育教学质量考核评估体系，从严整治教育“三乱”，坚决把学生和家长负担减下来。（20）进一步解决看病难问题，推动二、三级医院优质医疗资源下沉，建成“院办院管”社区卫生服务中心36家。将医保异地直接结算范围扩大到外出农民工和外来就业创业人员。打造健康医疗云平台，为市民提供便捷医疗服务。（21）着力解决看病贵问题，推行省药品集中采购、全药网跨区域采购“两网并行”模式，进一步降低药品价格。（22）推动市属医院走“精专特”发展之路，做强一批高水平专科医院。（23）依托养老云平台建设，提高养老助老服务能力，新增养老床位2000张，新建社区老年人日间照料中心和服务驿站30个。（24）做好特色文化传承与保护工作，依法保护修缮利用全市415处历史建筑，组建市芭蕾舞团和芭蕾舞学校，打造国际化哈尔滨交响乐团，力争两年内创作2至3部具有较高水准和市场感召力的原创高雅艺术作品。（25）丰富群众文化生活，整合利用大学、大厂、社会场馆资源，开展驻场演出和群众性文化活动1000场次以上。（26）开辟冰雪运动场所，浇建80处公益性室外滑冰场，每个区建设1个室内冰上活动中心。规划改造八区体育场和市冰上运动中心，实现主城区、县（市）城关镇住宅小区和开放式公园健身路径全覆盖。（27）建立应急管理新体制，建设城市应急指挥云平台系统。（28）提升消防能力，新建标准消防站和小型消防站32个。严厉打击小区场地私搭乱建和堵塞楼内外消防通道等违规违法行为，进一步消除火灾隐患。（29）建设城市安防云平台，安装视频监控设备4万个。（30）探索监管和服务新模式，在食品安全、生产安全、精准扶贫等领域试行保险公司介入新机制。

安全稳定的社会环境是广大人民群众安居乐业的基础。我们要进一步创新现代社会治理体系，不断提高社会治理社会化、法治化、智能化、专业化水平。严密防范暴恐活动，严厉打击敌对势力渗透破坏，严格落实地区和部门监管责任，持续提升平安哈尔滨建设水平。纵深推进扫黑除恶专项斗争，聚焦重点地区、重点行业领域，强力扫黑、铁腕惩恶、重锤打伞，彻底铲除黑恶犯罪滋生土壤。用好城市安全风险评估成果，落实安全生产主体责任，实施全过程安全管理，坚决防止重特大事故发生。整合市场监管领域监测检测资源，实施食品药品等方面有奖举报制度，加大食品、药品、产品质量和特种设备安全监管，切实保障群众身体健康和生命安全。加快城乡建设、产权证照、劳动社保等重点领域信访积案化解，更好维护群众合法权益和社会和谐稳定。探索国防动员新机制，深化双拥共建，妥善做好退役军人接收安置工作，落实好抚恤优待政策。

（七）全面加强自身建设，打造人民满意政府。空谈误国，实干兴邦。幸福是奋斗出来的，振兴是实干出来的。我们要把政府自身建设放在突出位置，着力锻造忠诚干净担当的政府公职人员队伍。

加强政治建设，始终保持对党绝对忠诚。坚持把党对一切工作的领导贯穿政府工作始终，从政府组成人员和工作部门做起，牢固树立“四个意识”，增强“四个自信”，坚决做到“两个维护”，真正把习近平新时代中国特色社会主义思想和习近平总书记关于东北振兴的重要论述，转化为推动哈尔滨全面振兴全方位振兴的生动实践。教育引导政府公职人员恪守职业道德，践行以人民为中心思想，切实做到诚信敬业、敢于担当、勇于担当、善于担当。以争创第六届全国文明城市为牵动，通过价值引领、典型带动和文化传承，引导广大市民自觉树立担当奉献、爱之深切的城市精神，为哈尔滨加快发展凝聚磅礴力量。

推动思想解放，进一步提升干事创业精气神。坚信越改革越有活力、越开放越能发展、越创新越有动力，深入开展解放思想推动高质量发展大讨论，更加注重向先进地区对标对表，从一点一滴做起，从关键少数抓起，从制约发展的陈规旧弊改起，着力打破计划经济体制形成的条条框框、思维定式和路径依赖，鼓励勇于探索、推陈出新，解决循规蹈矩、求稳畏难、生冷硬横、自我感觉良好等问题，在解放思想中发现问题、认清差距，用解放思想、深化改革来寻求解决问题的办法，以实事求是、善做善成的政治品格和聪明才智，推动哈尔滨早日实现腾飞梦想。

坚持不懈学习，不断提高政府工作专业水准和科学化决策能力。采取走出去、请进来等多种形式，高标准严要求教育引导，培养政府公职人员的专业思维、专业素养、专业方法，凭专业说话，按程序办事，不断提高工作落实效率和效益。结合经济运行云平台建设，强化科学精准研判，持续提升工作的战略性、系统性和针对性。坚持一切从实际出发，在正确时间做正确的事，既不能超出当下发展阶段搞面子工程、政绩工程，也不能囿于一时困难瞻前顾后、裹足不前。强化资金使用效益评估，及时引导和撬动社会资本助力发展。进一步转变工作作风，提高政府工作执行力，加强跟踪问效，把责任落实到位、压力传导到位、措施具体到位，以钉钉子精神和踏石留印、抓铁有痕的韧劲，推动各项决策落地生根、开花结果。

强化立体监督，切实做到依法行政廉洁从政从严治政。坚决执行人大及其常委会决议决定，自觉接受人大法律和工作监督、政协民主监督、媒体监督和公众监督。严守政治纪律、政治规矩和廉洁从政各项规定，严格落实中央八项规定精神，坚决防止新形式主义和官僚主义。树立过紧日子思想，勤俭节约，杜绝浪费，把政府有限资金用在刀刃上。始终保持正风肃纪高压态势，依法规范权力和资金运行，加大对经济责任、重大投资项目和民生工程的审计力度，把政府行为置于全方位监督之下，让每一名政府公职人员知敬畏、存戒惧、守底线，永葆为民务实清廉的公仆本色。

各位代表，我们正处在千帆竞发、百舸争流的新时代，推动哈尔滨全面振兴全方位振兴的任务艰巨、使命光荣、责任重大。让我们更加紧密地团结在以习近平同志为核心的党中央周围，在省委、省政府和市委的坚强领导下，紧紧依靠全市人民，解放思想、坚定信心、凝神聚力、真抓实干，共同创造哈尔滨更加美好的明天！
